# Access to Primary Care Clinics for Patients With Chronic Pain Receiving Opioids

**DOI:** 10.1001/jamanetworkopen.2019.6928

**Published:** 2019-07-12

**Authors:** Pooja A. Lagisetty, Nathaniel Healy, Claire Garpestad, Mary Jannausch, Renuka Tipirneni, Amy S. B. Bohnert

**Affiliations:** 1 Department of Internal Medicine, School of Medicine, University of Michigan, Ann Arbor; 2 Institute for Healthcare Policy and Innovation, Ann Arbor, Michigan; 3 Center for Clinical Management Research, Veterans Affairs Ann Arbor Healthcare System, Ann Arbor, Michigan; 4 Department of Psychiatry, School of Medicine, University of Michigan, Ann Arbor

## Abstract

**Importance:**

Opioid-prescribing policies and guidelines aimed at reducing inappropriate opioid prescribing may lead physicians to stop prescribing opioids. Patients may thus encounter difficulties finding primary care practitioners willing to care for them if they take opioids.

**Objectives:**

To assess practitioner willingness to accept and continue prescribing opioids to new patients with pain and whether this willingness differs across payer types.

**Design, Setting, and Participants:**

This survey study used a simulated patient call audit method. A brief telephone survey was administered to all clinics followed by a call using a patient script simulating an adult patient with chronic pain who was taking long-term opioids. The patient had Medicaid or private insurance. Calls were made between June 22 and October 30, 2018, to 667 primary care clinics that served a general adult population in Michigan. Clinics that accepted both Medicaid and private insurance, took new patient appointments, and were successfully recontacted for the simulated call were eligible for the study.

**Main Outcomes and Measures:**

Prevalence of clinics’ acceptance of new patients receiving prescription opioids overall and by clinic characteristics and insurance type.

**Results:**

Of the 194 eligible clinics, 94 (48.4%) were randomized according to insurance type to receive calls from research assistants posing as children of patients with Medicaid and 100 (51.5%) to receive calls from those with private insurance. Overall, 79 (40.7%) stated that their practitioners would not prescribe opioids to the simulated patient. Thirty-three clinics (17.0%) requested more information before making a decision. Compared with single-practitioner clinics, clinics with more than 3 practitioners were more likely (odds ratio [OR], 2.99; 95% CI, 1.48-6.04) to accept new patients currently taking opioids. No difference was found in access based on insurance status (OR, 0.92; 95% CI, 0.52-1.64) or whether the clinic offered medications for opioid use disorders (OR, 1.10; 95% CI, 0.45-2.69).

**Conclusions and Relevance:**

The findings suggest that access to primary care may be reduced for patients taking prescription opioids, which could lead to unintended consequences, such as conversion to illicit substances or reduced management of other medical comorbidities.

## Key Points

**Question:**

How likely are primary care practitioners to accept new patients taking prescription opioids for pain management, and does this differ based on insurance type?

**Findings:**

In this survey study of Michigan primary care clinics, 79 clinics contacted (40.7%) stated that their practitioners would not accept new patients receiving opioid therapy for pain. There was no difference based on insurance type.

**Meaning:**

The findings suggest that access to primary care may be reduced for patients taking prescription opioids, which could lead to unintended consequences, such as conversion to illicit substances or poor management of other mental and physical comorbidities.

## Teaser

This survey study of Michigan primary care clinics assesses practitioner willingness to accept and continue prescribing opioids to new patients with pain and whether this willingness differs across payer types.

## Introduction

Since the 2000s, the number of opioid-related overdoses has continued to increase.^1,2^ Many of these overdoses have been associated with increased use of prescription opioid analgesics.^3^ To reduce inappropriate opioid prescribing, several agencies, including insurance companies, have initiated policies that mandate clinical practices, such as mandatory checks of prescription drug monitoring programs and limits on dosages of opioids being prescribed and duration of opioid therapy.^4,5^ In Michigan, prescribers are mandated to check the prescription drug monitoring program before every new and old prescription; complete an Opioid Start Talking Form, which discusses the risks and benefits of opioids; and have a bona fide patient-prescriber relationship with the patient to initiate opioid therapy.^6^ In addition, the 2016 Centers for Disease Control and Prevention Guideline for Prescribing Opioids for Chronic Pain emphasizes using nonopioid therapies for chronic pain and trying to use the lowest effective dose if opioid therapy is indicated.^5^ Many of these guidelines and policies have achieved the desired result of reduced opioid prescribing.^7,8^ However, stakeholders have expressed concern that these new policies have led physicians to stop prescribing opioids completely, even to certain patients for whom the benefits of opioids may outweigh the risks.^9,10^

Patients may thus encounter difficulties finding primary care practitioners willing to care for them if they take opioids.^11^ Popular media outlets have described this population that is now being displaced from health care systems as *opioid refugees*.^12,13^ Primary care practitioners may still be willing to provide care for other medical issues, such as hypertension, but they may turn away new patients who need opioids for pain. A North Carolina Medical Board Survey, for example, found that 13% of 2262 practitioners surveyed had stopped accepting new patients with chronic pain who were taking opioids.^14^ This restriction may leave patients without options for slow opioid tapers; nonopioid treatment options; harm-reduction approaches, such as naloxone provision; or even screening and referrals for underdiagnosed opioid use disorders (OUDs). Many experts have expressed concern that abandoning this population could lead to unintended consequences, such as increased use of more potent illicit opioids or potentially even increased risk of suicide.^15^ However, thus far to our knowledge, there have been no quantitative studies to fully assess the scope of this phenomenon.

This study used simulated patient calls (ie, calls from research assistants posing as children of patients) to assess practitioner willingness to accept new patients who are currently undergoing long-term opioid therapy. The calls were structured to allow for different clinic-level practices on addressing the patient’s opioid use (eg, continuing opioid prescribing, case-by-case evaluation, and offering tapers after initial meetings). We also examined whether clinics would be more willing to provide care if the patient had private vs Medicaid insurance and by whether the clinic provides OUD treatment.

## Methods

This survey study used an audit method described in previous studies.^16,17^ We obtained a list of 4850 Michigan primary care practitioners from a commercial database of office-based practitioners that was last updated in 2014 and described in a previous study.^17^ This database includes a wide range of practices ranging from academic practices to safety-net clinics.^18^ We randomly selected 667 Michigan primary care clinics stratified by practice size (1-3 or >3 practitioners) and called these clinics between June 22 and October 30, 2018. The University of Michigan Institutional Review Board deemed this study not regulated because it did not collect individual information about clinic patients or staff. The American Association for Public Opinion Research (AAPOR) reporting guideline was followed when reporting response rates for the survey and the simulated patient script.

We surveyed clinics by telephone about the number and type of practitioners (eg, physicians, nurse practitioners, and physician assistants), insurances accepted, appointment availability, and whether their practitioners use medications to treat OUDs (eAppendix 1 in the Supplement). We classified a clinic as a community health clinic if it was listed on the Health Resources and Services Administration website as a community-based clinic that delivers care to the “nation’s most vulnerable individuals and families.”^19^ We determined whether a clinic was rural or urban using data from the US 2010 Population Density Data.^20^ If the population density was more than 1000 people per square mile, the clinic was deemed to be urban.^21^ Clinics accepting new patients with both Medicaid and private insurance were deemed eligible for inclusion and received a second call from a simulated patient randomized to having Medicaid or Blue Cross Blue Shield insurance. This method ensured that none of the denials that we received were because the clinic was not accepting new patients or the simulated patient’s insurance. A prior study^15^ on general primary care access for Medicaid patients in Michigan reported a 54% acceptance rate. We assumed that needing opioids for chronic pain may result in a 20% reduction in access to primary care visits and that this reduction would be a clinically significant reduction in access. Therefore, assuming 30% of Medicaid patients would be accepted, the minimum required sample size was 93 clinics per group (N = 186) to detect a 20% difference with 80% power and α = .05.

Four trained research assistants (RAs) (including C.G., N.H.) used a standardized script (eAppendix 2 in the Supplement) to pose as the child of an adult woman who needed a new primary care appointment. The lead RA (C.G.) was involved in the study and script design and trained the second lead RA (N.H.). These 2 RAs then trained the remaining assistants and conducted periodic audits to gather feedback on where there may be problems. All RAs were trained to follow the script exactly as written, leaving little to no room for variability among callers.

We chose to use a scenario with the relative of the patient to avoid having to reveal any identifying details, such as Social Security numbers and birthdates, that patients would know about themselves but could plausibly not be known by their children. Posing as a child of the patient also helped eliminate sex differences among the RAs. We were also aiming to simulate a scenario of an older adult requiring long-term opioid treatment for chronic pain and not a younger individual needing opioids who may have a higher likelihood of being perceived as a patient misusing opioids. In this script, RAs revealed the patient’s health insurance and asked, “Before we get too far, is it okay if my mother takes opioids for pain?” The RAs documented each clinic’s willingness to provide primary care. If the clinic requested more information, the RAs stated that the patient was taking two to three 5-mg oxycodone hydrochloride and acetaminophen (Percocet) tablets per day for back pain after a remote car accident, as well as lisinopril and simvastatin. We did not probe whether practitioners would have been willing to schedule the patient for non–pain-related issues because we were not confident that a scheduler would be able to accurately answer this question without practitioner input.

### Statistical Analysis

Clinic characteristics were summarized via descriptive statistics. Generalized logistic regression models estimated the odds of acceptance of a new patient currently taking opioids by insurance type, clinic size, and availability of OUD medications. Analyses used SAS version, 9.4 (SAS Institute Inc).

## Results

**Figure 1.**
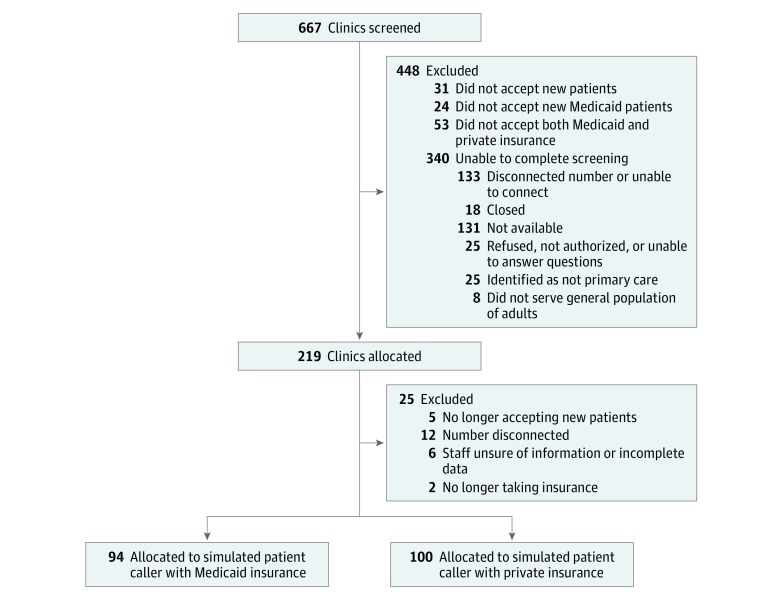
Flow Diagram of Clinic Allocation

**Figure 2.**
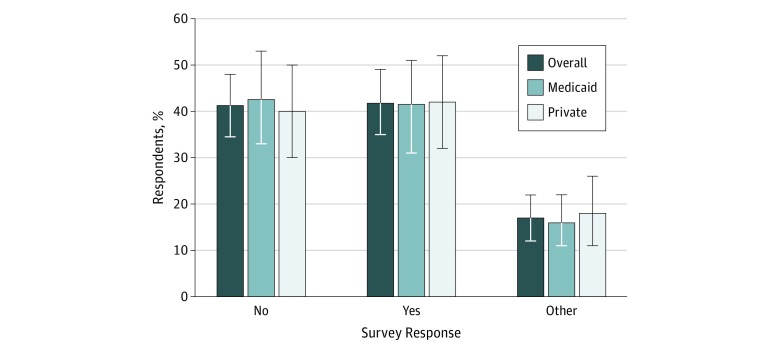
Percentage of 194 Clinics Accepting New Patients Currently Taking Opioids Error bars indicate 95% CIs.

Of the 667 clinics screened, 219 (32.8%) were eligible for study inclusion. Of the 219 eligible clinics, 194 (88.6%) completed the scripted call (Figure 1). Ninety-four clinics (48.4%) were allocated to simulated patient calls with Medicaid and 100 (51.5%) to Blue Cross Blue Shield (Figure 1). Of these clinics, 79 (40.7%) stated that their practitioners were not willing to provide care for new patients taking opioids. A total of 81 clinics (41.8%) were willing to schedule an initial appointment (Figure 2). An additional 33 clinics (17.0%) requested more information before making a decision. After receiving this information, 1 clinic accepted the patient, 4 did not accept the patient, 20 stated that the practitioner would decide about opioid prescribing after the first visit, 7 stated that they would refer the patient to a pain clinic, and 1 requested faxed medical records.

Compared with single-practitioner clinics, clinics with more than 3 practitioners were more likely (odds ratio [OR], 2.99; 95% CI, 1.48-6.04) to accept new patients currently taking opioids (Table). In addition, clinics that were community health centers were more likely to accept new patients currently taking opioids (OR, 3.10; 95% CI, 1.11-8.65). No difference was found in primary care practitioner acceptance based on insurance status (OR, 0.92; 95% CI, 0.52-1.64), whether the clinic offered OUD treatment (OR, 1.10; 95% CI, 0.45-2.69), or whether the clinic was urban (OR, 0.65; 95% CI, 0.37-1.16).

**Table.** Characteristics of Clinics Accepting New Patients Currently Taking Opioids

Characteristic	Clinics (N = 194)^a^	OR (95% CI) for Acceptance of New Patients Currently Taking Opioids
Urban or rural status		
Urban	103 (53.1)	0.65 (0.37-1.16)
Rural	91 (46.9)	1 [Reference]
Community health center^b^	18 (9.3)	3.10 (1.11-8.65)
Insurance type		
Medicaid	94 (48.4)	0.92 (0.52-1.64)
BCBS	100 (51.5)	1 [Reference]
No. of practitioners, median (IQR)^c^	3 (1-5)	1.16 (1.05-1.30)
Grouped by clinic size		
1 Practitioner	59 (30.4)	1 [Reference]
2-3 Practitioners	53 (27.3)	1.43 (0.68-3.02)
4-6 Practitioners	51 (26.3)	2.59 (1.19-5.67)
≥7 Practitioners	31 (16.0)	3.41 (1.31-8.84)
Practitioners prescribe MAT		
Yes	23 (11.8)	1.10 (0.45-2.69)
No	92 (47.4)	1 [Reference]
Don’t know	79 (40.7)	1 [Reference]

Abbreviations: BCBS, Blue Cross Blue Shield; IQR, interquartile range; MAT, medication-assisted treatment; OR, odds ratio.

^a^ Data are presented as number (percentage) of clinics unless otherwise indicated.

^b^ Community health center defined by Health Resources and Services Administration.

^c^ Includes physicians, nurse practitioners, and physician assistants.

## Discussion

A total of 40.7% of clinics were not willing to schedule an appointment for a new patient who was currently taking opioids for chronic pain. Insurance status and whether the clinic provided medication for treatment of OUD were not associated with willingness to accept the new patient currently taking opioids. However, larger clinics with more practitioners and community health centers were more willing to accept new patients currently taking opioids.

Our findings showed a higher prevalence of practices not willing to accept new patients than found by the previously referenced North Carolina Medical Board Survey (13%).^14^ This higher number of practices unwilling to provide care in our study may be associated with decreased social desirability bias using an audit method,^22^ regional differences, or the increasing numbers of practitioners refusing to treat patients taking opioids for pain. These findings may also reflect practitioners' discomfort with managing opioid therapy for chronic pain or treating patients with OUD as a result of pressures to decrease overall opioid prescribing.^9^ In addition, the findings may reflect frontline staff bias against what may be perceived as drug-seeking behavior and may not actually indicate prescriber decision-making or clinic-level policies. Future studies should evaluate whether different patient scenarios for requesting opioids result in different acceptance rates. However, regardless of the reason for denial, our results suggest that there are significant barriers in accessing primary care for patients taking opioids for chronic pain.

Our results also did not detect a difference in new patient appointment access for patients taking opioids based on insurance type alone. These findings differ from prior studies^23,24^ in the general population, which have indicated decreased access to primary care for patients covered by Medicaid compared with those with private insurance. These findings could suggest that clinic practices are more likely to be influenced by recent changes in opioid-prescribing policies than differences in reimbursement. In our study, larger clinics and community health centers were more willing to accept new patients taking opioid medication. These clinics may have more resources^25,26^ to care for this population and follow new time-consuming policies, such as checking state prescription drug monitoring programs.^25^ In addition, compared with single-practitioner clinics, practitioners in larger group practices may benefit from peer-to-peer practitioner support, which could potentially decrease stress and burnout associated with caring for this population.^27^

Buprenorphine is increasingly being used to manage chronic pain and OUD.^28,29^ Our study found that a low number of clinics provided any medications for treatment for OUD, and a large number of front-desk staff at the clinics that were called (79 [40.7%]) did not know whether their clinic offered OUD treatment. This finding generally highlights a lack of knowledge about treatment options and availability that should be further investigated. Moreover, future studies should evaluate whether increasing practitioner knowledge and use of buprenorphine could potentially increase access to treatment not only for patients with OUD but also for those taking long-term opioids for chronic pain. Practitioners familiar with prescribing buprenorphine may be more prepared to address potential OUD if the initial or future patient assessment indicates any signs of misuse.

Prior studies^30,31,32,33^ estimate that less than 10% of patients undergoing long-term opioid therapy develop OUDs. For this minority population, reluctance to schedule appointments for new patients may limit chances to screen for OUD, initiate primary care–based treatment with medications for OUD, and/or refer patients to addiction treatment. Furthermore, it may lead to unintended adverse health outcomes, including increased transition to illicit opioid use and reduced access to treatment for other medical and psychiatric comorbidities.^34^ There are likely many reasons behind this restriction in access, including individual practitioners’ bias or stigma against this patient population, increased pressures to reduce prescribing, and the lack of systems in place to support practitioners who may be willing yet are not able. Improving practitioner and clinic staff education on this issue could help reduce physician and front-line staff bias. In addition, support networks for practitioners who treat chronic pain or improved access to and coverage of nonpharmacologic pain treatments may ameliorate the presumed burden of taking on this patient population. Future policies will need to break down these barriers in access to care not only for patients with OUD but also for those with chronic pain for whom the benefits of prescription opioid therapy may outweigh the risks.

### Limitations

Study limitations include a single-state sample, which may reduce generalizability. We also excluded a large number of clinics because of inability to complete the screening call for the variety of reasons outlined in Figure 1. A previous secret shopper study^15^ had similar limitations. In addition, we did not probe whether practitioners would have been willing to provide primary care outside prescribing opioids because we were not confident that schedulers would be able to answer this question without practitioner input. However, even if the clinic was willing to see the patient for other primary care reasons, restrictions on scheduling appointments for patients taking opioids may leave patients with chronic pain and physical dependence on opioids searching for care in unforeseen situations, such as if their current prescriber retires or if the patient moves. Furthermore, such restrictions may decrease opportunities to perform patient-centered dose tapers or initiate nonopioid pain treatments. Also, our interaction with frontline clinic staff may or may not accurately reflect prescriber practices if the staff member was unaware of prescriber preferences. However, the first-line interaction simulated in this study accurately reflects patient experience in seeking care for chronic pain and therefore is a valid investigation into barriers patients may experience when accessing primary care for chronic pain.

## Conclusions

The findings suggest that access to primary care may be reduced for patients taking prescription opioids, which could potentially lead to unintended consequences, such as conversion to illicit substances or reduced management of other medical comorbidities.

## Notes

Supplement. **eAppendix 1. **Exhibit 1: Supplemental Exhibit 1 **eAppendix 2. **Exhibit 2: Patient Script
