# Supplementary material for: Access to Primary Care Clinics for Patients With Chronic Pain Receiving Opioids
Source: JAMA Netw Open. 2019 Jul 12;2(7):e196928. doi: 10.1001/jamanetworkopen.2019.6928 (PMC6628590; doi:10.1001/jamanetworkopen.2019.6928)
Supplement: Supplement. — eAppendix 1. Exhibit 1: Supplemental Exhibit 1 eAppendix 2. Exhibit 2: Patient Script [file jamanetwopen-2-e196928-s001.pdf]

Supplementary Online Content

Lagisetty PA, Healy N, Garpestad C, Jannausch M, Tipirneni R, Bohnert ASB. Access to primary care clinics for patients with chronic pain receiving opioids. *JAMA Netw Open*. 2019;2(7):e196928. doi:10.1001/jamanetworkopen.2019.6928

eAppendix 1. Exhibit 1: Supplemental Exhibit 1

eAppendix 2. Exhibit 2: Patient Script

This supplementary material has been provided by the authors to give readers additional information about their work.

© 2019 Lagisetty PA et al. *JAMA Network Open*.

eAppendix 1. Exhibit 1: Supplemental Exhibit 1

INTRO

Q1.1 Hi, are you located at … [READ STREET ADDRESS WITHOUT CITY AND STATE] INPUT INTO REDCAP TRACKER

IF MOVED, ask for:

New street address ___________________________________

New city, state, zip ___________________________________

> Put **dashes** in the number and include area code.
>
> Use the format ###-###-####

Q1.2 Is this phone number the main appointment line for primary care patients?

- Yes, main PC appointment line for above address (1) GO TO CLINIC INFORMATION SECTION (PAGE 2)
- No, main PC appointment line is ___________________________ (2) GO TO CLINIC INFORMATION SECTION (PAGE 2)
- Wrong number for address – no new number available → mark screener as **incomplete and track in tracking survey.**

General Instructions:

We are trying to establish whether or not this is a clinic location that includes adult outpatient primary care and to collect the main appointment line for that clinic.

Q1.1 - You need to check the clinic street address. We want the address where a patient goes for care, NOT a mailing address for billing or administrative matters. If the address needs to be corrected, enter the full correct address, NOT just the parts that need to be changed.

Q1.2 - You also need to check the clinic appointment line. Because clinics may have many phone numbers associated with them, we are focusing on obtaining the best phone number a patient would call to schedule a primary care appointment at the clinic location. If the person you speak with cannot give you an appointment line for the listed clinic address, a good phone number that connects to the listed clinic address is your next choice.

© 2019 Lagisetty PA et al. *JAMA Network Open*.

CLINIC INFORMATION SECTION

Thank you. I'm calling from the University of Michigan to collect basic information about primary care clinics across the state Do you have time to answer a few quick questions? First…
(If they try to forward you onto a larger corporation (such as one outside of Michigan), push that you are calling locally and the questions are fairly straightforward.)

Q2 Does the clinic serve a general population of adults or is it only for a specific group like children, the elderly, students, veterans or Native Americans?

- General population of adults (1)
- Other (Children only, Elderly only, Students only, Veterans only, Native Americans only, etc.) (0)
  If "Other", END CALL and thank them for their time.

General Instructions:

It is important NOT to hesitate between the introduction and the first question.

If this clinic serves only children, only elderly, veterans, Native Americans, or students enrolled in a specific institution, then it is **out of scope**, and this should take you to the end of the call. OB/Gyn clinics are also **out of scope**. Clinics that serve adults AND children are fine (e.g. Family Medicine, Medicine/Pediatrics).

CLINIC INFORMATION SECTION – PAGE 2 OF 4

We only want counts of the primary care providers who see adult patients. Do not include pediatricians (who see only children) or specialists who may see patients at the same clinic.

If you are speaking with somebody at a different location from that listed, confirm you are asking about only the listed location.

Q3. How many total providers at the clinic (such as doctors, nurse practitioners or physician assistants) serve as primary care providers for adults? [If you don't know the exact number, an estimate is OK. Are there any? One or more? More than 10, less than 10?]

Enter numbers of current PCPS at the time of the call, even if this will change soon.

MDs (medical doctors) and DOs (doctors of osteopathy) get counted in the "Doctor" category. All other PCPs get counted in the "other PCPs" category, except residents who

© 2019 Lagisetty PA et al. *JAMA Network Open*.

Q3.1. Of that total, how many providers are MD or DO?

____

Q3.2 Of your total providers, how many are NPs?

____

DEFINITION IF NEEDED

[A primary care provider would be an MD, a DO, or others like nurse practitioners or physician assistants who can provide basic medical care for adults, prescribe medication, and manage their referrals to specialist doctors. For our purposes, this would not include chiropractors, or practitioners of acupuncture, homeopathic, holistic or other forms of non-medical treatment.]

Q4. Do your primary care providers accept any Blue Cross Blue Shield Insurance Plans?

- No (0)
- Yes (1)
- Refused (2)
- Don't know (3)

Q5. Do your primary care providers accept Medicaid?

- No (0)
- Yes (1)
- Refused (2)
- Don't know (3)

Q6. [If Yes to Q5] Do your providers take Medicaid managed care through:

Meridian Health Plan of Michigan

McLaren Health Plan

UnitedHealthcare Community Plan

Molina Healthcare of Michigan

Aetna Better Health of Michigan

Priority Health Choice

Stop reading when they accept a plan and write <u>one</u> specific plan they accept or indicate unknown if they cannot name a specific plan: ___________________________

Q7. Are any of your primary care providers in your clinic accepting NEW patients?

- Not accepting new patients (0)
- Accepting new patients (1)
- Refused (2)
- Don't know (3)

Q8 [IF YES TO Q7] Is that for all insurance plans?

- Not accepting new Medicaid patients (0)
- Accepting new Medicaid patients (1)
- Don't know (4)

© 2019 Lagisetty PA et al. *JAMA Network Open*.

- Accepting new private insurance patients only (2)
- Accepting new pts across all insurance types (3)
- Other (5)

Q9. Do you know if any of your providers prescribe Vivitrol (naloxone) or Suboxone (buprenorphine) for patients with Opioid Use Disorders or Addiction?

- No (0)
- Yes (1)
- Refer out to specialist (2)
- Don't know (3)
- Refused (4)

Q10. [IF YES TO Q9], which medications?

- Suboxone (Buprenorphine) (0)
- Vivitrol (naloxone) (1)
- Both (2)
- Don't know specifics (3)

Make it clear we are NOT directing any Medicaid or OUD patients to them.

Thank them for their time. END OF CALL

Add anything that unique/interesting comes up throughout call in comments section next to the question.

© 2019 Lagisetty PA et al. *JAMA Network Open*.

eAppendix 2. Exhibit 2: Patient Script

Screening calls script

Q1: Hi, are you currently taking new patients?

- No (0) (Thank them for time) and enter in Notes
- Yes (1)

Q2: [If Yes]. Oh good, I wanted to see if I could set up a primary care appointment for my mom. She has XXXXXX (Medicaid or BCBS) insurance, is that okay? (based on randomization post screener)

a. No (0)
b. Yes (1)
c. Only some of our providers accept Medicaid (3) (document specifics in free text box)
d. Other (4)

Q3. Respondent may then ask for other information about the patient such as Patients name, address, age. Prior to giving this information out: Say "Before, we get too far- I just wanted to let you know that she takes opioids for pain. Is that going to be okay with your providers?" (Document verbatim response in free text box)

a. No, none of our providers prescribe opioids (0)
b. Yes, that is okay (1)
c. Some, but not all, of our providers prescribe opioids (2) (document specifics in free text box)
d. I will have to ask our provider and get back to you. (3)
e. We need more information. (4)

QX: If (some providers and not others): Is there a reason only some providers are able to prescribe opioids? (add specifics in comment box)

a. Insurance reasons
b. Licensing regulations (e.g. our NP cannot prescribe opioids)
c. Provider preference
d. Don't know
e. Other

Q4. If A (no): "Oh okay, that is too bad, are there any exceptions?" Document verbatim response in free text box.

Q5. If B, C or D (yes, or some version of yes)– "Great, thanks for your time. I'll have my mom call back to make the appointment herself".

Q6: If F. (need more information): "what type of information do you need?"

© 2019 Lagisetty PA et al. *JAMA Network Open*.

Provide the following information as prompted: "Name: Mary Smith. Her birthdate is January 26th, 1960-something, oh shoot I forget the year, I'll have to get back on you that. She has high blood pressure and high cholesterol Her medications are 20mg of Lisinopril, 20mg of simvastatin and I think she takes about 2-3 5mg Percocet a day, for lower back pain from a car accident 7 years ago. What information did the clinic want to collect: Check all that apply :

a. Opioid type (1)
b. Opioid dose (2)
c. Reason for Opioid prescription (3)
d. Time on opioids (4)
e. Other medications (5)
f. Other comorbidities (6)
g. On disability (7)
h. Birthdate or other demographics (8)
i. Other (document in text box) (9)

Q7. If they need more information, what was the result? If a call back is needed, call 248-387-5230 (google voice account).

a. Patient was denied immediately (0)
b. Patient was accepted immediately (1)
c. Patient was accepted after a call back (2)
d. Patient was denied after a call back (3)
e. Other (document in comment box)

Q8: Regardless of the above outcome, wrap up the conversation by saying: "Thank you for your time. I will have my mom call back to schedule the appointment based on her schedule." OR "Thanks anyways for your time." <u>END CALL</u>

© 2019 Lagisetty PA et al. *JAMA Network Open*.
